# Epidemiology of the non-head and neck sebaceous carcinoma and implications for distant metastasis screening

**DOI:** 10.3389/fonc.2024.1395273

**Published:** 2024-05-10

**Authors:** Xi Chen, Yangyang Hao, Mengwei Chou, Jianqiang Yang

**Affiliations:** Department of Dermatology, The First People’s Hospital of Huzhou, Huzhou, Zhejiang, China

**Keywords:** sebaceous carcinoma, non-head and neck, distant metastasis, SEER, overall survival, disease-specific survival

## Abstract

**Introduction:**

Extraocular sebaceous carcinoma (SC), particularly those outside the head and neck region, is rare and not well-described.

**Purpose:**

This study aimed to explore the epidemiology and identify the prognostic factors of non-head and neck SC, describe the possible relevant factors of distant metastasis, and provide implications for distant metastasis screening.

**Methods:**

Data from the 17 registries in the Surveillance, Epidemiology, and End Results database were retrospectively collected for patients with SC outside the head and neck from 2000 through 2020. Overall survival (OS) and disease-specific survival (DSS) were the primary endpoints. Survival analysis was conducted through Kaplan–Meier curves, and multivariate analysis was carried out using Cox proportional hazard models.

**Results:**

A total of 1,237 patients with SC outside the head and neck were identified. The mean age at diagnosis of the entire patient cohort was 67.7 years (30 to 90+ years), and the mean tumor size was 2.2 cm (0.1–16 cm). Patients with distant disease experienced the lowest OS (mean, 29.5 months) than those with localized disease and regional disease (*p* < 0.0001). Multivariate analysis revealed that age, tumor size, and stage were independent determinants of OS; age, stage, and primary site were independent determinants of DSS. Tumor grade and lymph node status had less prognostic value for survival. Undifferentiated tumors have a trend toward distant metastasis, especially those at the primary site of the trunk.

**Conclusion:**

The prognosis of the non-head and neck SC is excellent, while the survival of distant disease is very poor. Distant metastasis screening can be considered for undifferentiated tumors, especially those located in the trunk region with large tumor sizes.

## Introduction

Sebaceous carcinoma (SC) is a rare cutaneous malignancy that arises from the sebaceous gland, one of the adnexal structures of the skin (1). SC most commonly affects the head and neck, especially the periocular region; moreover, it can occur in many other locations of the body, such as the external genitalia, trunk, and extremities (2). Sebaceous carcinoma is generally considered indolent, but there is also the possibility of local infiltration and distant metastasis (3). Guidelines for the management of SC, including screening and identification for distant metastasis, are currently scarce.

Given the low incidence of SC outside the head and neck, most of the records are limited to case reports with short follow-ups. The epidemiology regarding this malignancy is thus scanty. To date, this study presents the largest population-based multivariate analysis to determine prognostic factors for patients diagnosed with SC, specifically outside the head and neck via the Surveillance, Epidemiology, and End Results (SEER) program database. Moreover, this analysis provides important counseling value about distant metastasis screening for non-head and neck SC in clinical practice.

## Methods

### Patient selection

The SEER database’s 17 registries were queried for patients diagnosed with SC outside the head and neck between the years 2000 and 2020. The right to access these data through SEER Stat software was granted by the National Cancer Institute upon submission of a signed SEER data use agreement. This population-based database contains comprehensive data on cancer survival and is updated annually by the National Cancer Institute. It comprises 26% of the population in 17 geographic regions of the USA. Patients diagnosed with SC outside the head and neck were identified using the International Classification of Diseases for Oncology (ICD-O) histologic code 8410/3 (sebaceous carcinoma) and site-specific codes C44.5 (skin of trunk), C44.6 (skin of upper limb and shoulder), C44.7 (skin of lower limb and hip), C51.0 (labium majus), C51.1 (labium minus), C51.9 (vulva, NOS), C60.2 (body of penis), C60.9 (penis, NOS), and C63.2 (scrotum, NOS).

### Variables

The following primary data were extracted for analysis: sex, age at diagnosis, race, primary site, tumor size, lymph node involvement (yes/no), tumor grade (well differentiated, moderately differentiated, poorly differentiated, and undifferentiated), stage (localized, regional, and distant), cause of death, survival months, and vital status. Tumor grade was categorized as low (well to moderately differentiated) or high (poorly differentiated or undifferentiated). Race was grouped into three categories, including white, black, and others (American Indian/Alaska Native, Asian, or Pacific Islander). For the purpose of analysis, the continuous variables age and tumor size were transformed into categorical variables. Age was presented as four subgroups (years): ≤ 60, 61–70, 71–80, or > 80, and tumor size was categorized into three subgroups (cm): ≤ 2, 2–4, or > 4. In addition, the anatomic location of C51.0, C51.1, C51.9, C60.2, C60.9, and C63.2 were reclassified as external genitalia. Treatment modality was initially collected but was not reported due to large deficiencies in radiotherapy data.

### Statistical analysis

The primary endpoints were defined as the time in months from diagnosis to death from any cause for overall survival (OS) and as the time from diagnosis to a documented death due to SC for disease-specific survival (DSS). Kaplan–Meier survival analysis was conducted to assess the impact of various variables on OS and DSS, and the statistical difference in curves was calculated by log-rank tests. All clinically significant variables or covariates with a log-rank *p* < 0.2 on univariate analysis were chosen for multivariate analysis. Through Cox proportional hazard models, multivariate analysis was performed to identify prognostic factors. All statistical analyses were carried out using SPSS version 26.0. Statistical significance in this study was set at two-sided *p* < 0.05.

## Results

### Baseline characteristics

A total of 1,237 patients diagnosed with SC outside the head and neck from 2000 through 2020 were identified and included in the analysis. The demographics and clinicopathologic characteristics are listed in **Table 1**. There was a predominance of men (66.5%) and whites (91.2%). The mean and median ages at diagnosis of the entire patient cohort were 67.7 years and 68 years, ranging from 30 to 90+ years (90+ is regarded as 90 in statistical analysis). Among the patients with known tumor stage and grade, 96.2% had localized disease, and 79.1% presented with low-grade features. Precise tumor sizes were acquired for 548 cases (mean, 2.2 cm), of which 33% have diameters greater than 2 cm with a maximum size of 16 cm. The mean tumor size of patients with distant metastasis was 7 cm (range, 2–15.5 cm). The overall rate of lymph node involvement was 1.2%. In this cohort, the most common primary site was the trunk (65%), followed by the upper limb and shoulder (23.8%), the lower limb and hip (7.6%), and the external genitalia (3.6%).

**Table 1:** The demographics and clinicopathologic characteristics of patients diagnosed with sebaceous carcinoma outside the head and neck.

Characteristics	*N* (%)
Age (years)	
≤ 60	379 (30.6%)
61–70	329 (26.6%)
71–80	296 (23.9%)
> 80	233 (18.8%)
Sex	
Male	822 (66.5%)
Female	415 (33.5%)
Race	
White	1,073 (91.2%)
Black	48 (4.1%)
Others	56 (4.8%)
Tumor size (cm)	
≤ 2	367 (67.0%)
2–4	114 (20.8%)
>4	67 (12.2%)
Primary site	
Trunk	804 (65.0%)
Upper limb and shoulder	294 (23.8%)
Lower limb and hip	94 (7.6%)
External genitalia	45 (3.6%)
Grade	
Low (well to moderately differentiated)	257 (79.1%)
High (poorly differentiated or undifferentiated)	68 (20.9%)
Summary stage	
Localized	876 (96.2%)
Regional	20 (2.2%)
Distant	15 (1.6%)
Lymph node involvement	
Yes	10 (1.2%)
No	852 (98.8%)

### Survival analysis by sex, race, and age

**Figure 1:**
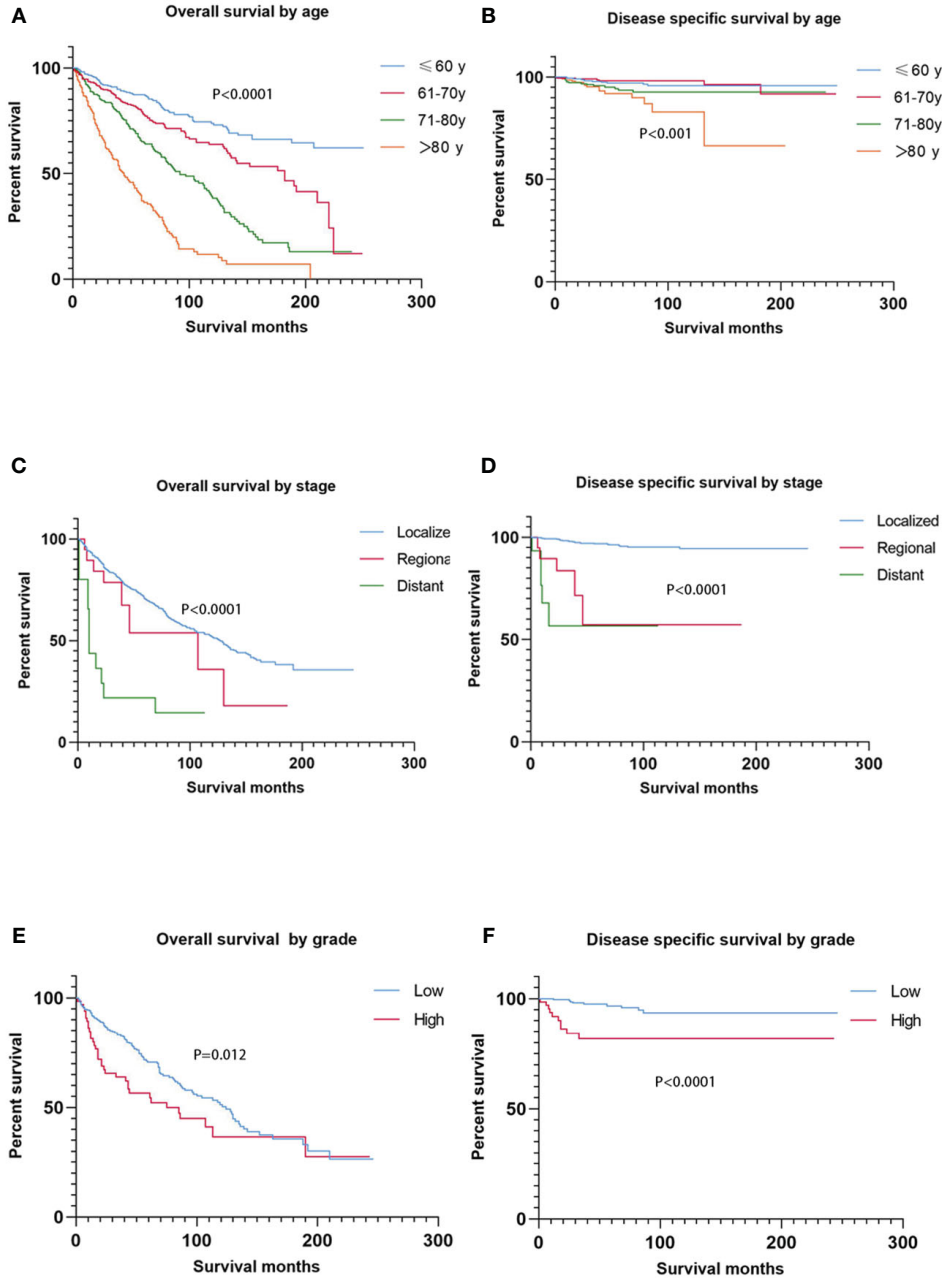
Kaplan–Meier estimates of OS and DSS by age, stage, and grade. OS decreased as age increased **(A)**, and patients over 80 years had significantly worse DSS than other age groups **(B)**. Patients with distant disease experienced the lowest OS **(C)**, and patients with localized disease experienced the best DSS **(D)**. Regarding grade, patients with high differentiation had better OS and DSS than those with low differentiation **(E**, **F)**. OS, overall survival; DSS, disease-specific survival.

No statistical significance of survival analysis in OS and DSS was reached with regard to gender and race (*p* > 0.05). This cohort spanned a wide age range, from 30 to 90+, and cases were relatively evenly distributed across the four age subgroups (≤ 60, 61–70, 71–80, > 80). OS decreased as age increased (*p* < 0.0001), and patients over 80 years of age had significantly worse DSS than other age groups (*p* < 0.001) (**Figure 1**).

### Survival analysis by tumor stage and grade at diagnosis

Survival analysis from Kaplan–Meier curves revealed statistical significance in the tumor stage (**Figure 1**). In this study, 911 cases with a known SEER stage were available, and only 15 patients had distant disease (1.6%), experiencing the lowest 5-year OS of 21.8% (mean OS, 29.5 months). Of the 15 patients with distant disease, seven had specific tumor grades, and most (six of seven) were characterized by poorly differentiated or undifferentiated histological morphology. Patients with localized disease had statistically better DSS than those with regional disease and distant disease (*p* < 0.0001). In addition, patients with low-grade tumors experienced better survival than those with high-grade tumors, regardless of OS (*p* = 0.012) or DSS (*p* < 0.0001) (**Figure 1**).

### Survival analysis by tumor size, anatomic site, and lymph node status

**Figure 2:**
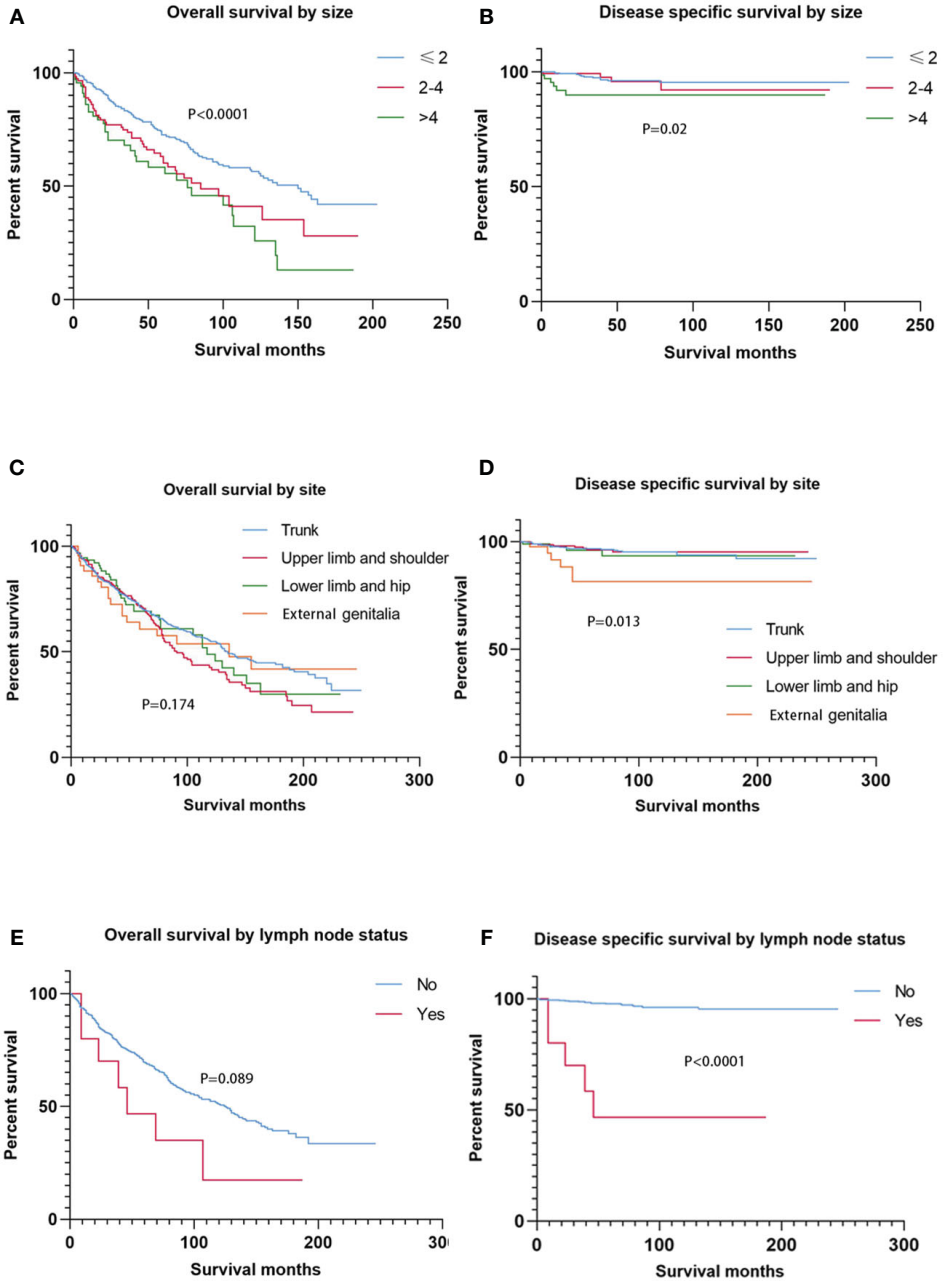
Kaplan–Meier estimates of OS and DSS by tumor size, primary site, and node status. Patients with tumors ≤ 2 cm had better OS than those with tumors > 2 cm (2–4 cm and > 4 cm), and patients with tumors > 4 cm had the worst DSS **(A**, **B)**. The primary site had no prognostic value for OS, while SC located on the external genitalia had worse DSS than those of other sites. There was no significant difference in OS between node-negative cases and node-positive cases, while node-positive cases experienced higher DSS than node-negative cases. OS, overall survival; DSS, disease-specific survival; SC, sebaceous carcinoma.

Smaller tumor size (≤ 2 cm) at presentation was associated with better OS than greater tumor size (2–4 cm and > 4 cm) (*p* < 0.0001). In addition, patients with tumors larger than 4 cm had the worst DSS (*p* = 0.02) (**Figure 2**). No significant difference in OS was found among anatomic sites (*p* = 0.174), while SC located on the external genitalia had worse DSS than those of other sites (*p* = 0.013) (**Figure 2**). Notably, patients with lymph node involvement had significantly worse DSS than those without lymph node involvement (*p* < 0.0001). On the contrary, lymph node involvement or not had less prognostic value in OS (*p* = 0.089) (**Figure 2**).

### Multivariate analyses on OS and DSS

**Tables 2**, **3** show the multivariate analyses of OS and DSS, respectively. Increasing age and tumor size were independent and significant predictors of worse OS (*p* < 0.0001). Moreover, the SEER stage at “distant” increased the risk of overall mortality than those at “localized” (HR, 6.97 [95% CI, 3.71–13.10]). Age at “71–80 years” (HR, 3.02 [95% CI, 1.32–6.93]) and “80+ years” (HR, 4.89 [95% CI, 2.10–11.37]) had worse DSS than those ≤ 60 years. Compared to patients with localized disease, patients with distant disease experienced significantly higher DSS (HR, 23.62 [95% CI, 6.68–83.49]). In addition, the primary site was a statistically significant predictor of DSS, and the SC located on the external genitalia had a 3.3 times greater risk of death than those on the trunk.

**Table 2:** Multivariate analyses of overall survival for nonhead and neck sebaceous carcinoma.

Characteristics	HR	95% CI	*p*-value
Age			
≤ 60	Reference		
61–70	1.89	1.40–2.55	0.000
71–80	3.76	2.83–5.00	0.000
> 80	8.37	6.27–11.19	0.000
Tumor size (cm)			
≤ 2	Reference		
2–4	1.93	1.37–2.71	0.000
> 4	2.87	1.88–4.39	0.000
Primary site			
Trunk	Reference		
Upper limb and shoulder	1.17	0.95–1.45	0.143
Lower limb and shoulder	0.99	0.69–1.40	0.934
External genitalia	0.95	0.60–1.50	0.816
Summary stage			
Localized	Reference		
Regional	1.16	1.16–0.44	0.763
Distant	6.97	3.71–13.10	0.000
Grade			
Low (well to moderately differentiated)	Reference		
High (poorly differentiated or undifferentiated)	1.10	0.73–1.65	0.647
Lymph node involvement			
No	Reference		
Yes	0.58	0.205–1.65	0.310

**Table 3:** Multivariate analyses of disease-specific survival for nonhead and neck sebaceous carcinoma.

Characteristics	HR	95% CI	*p*-value
Age			
≤ 60	Reference		
61–70	1.37	0.52–3.56	0.524
71–80	3.02	1.32–6.93	0.009
> 80	4.89	2.10–11.37	0.000
Tumor size (cm)			
≤ 2	Reference		
2–4	1.93	0.43–3.82	0.659
> 4	1.07	0.31–3.65	0.913
Primary site			
Trunk	Reference		
Upper limb and shoulder	0.80	0.38–1.71	0.571
Lower limb and hip	1.17	0.40–3.42	0.775
External genitalia	3.32	1.34–8.26	0.010
Summary stage			
Localized	Reference		
Regional	4.29	0.96–19.14	0.056
Distant	23.62	6.68–83.49	0.000
Grade			
Low (well to moderately differentiated)	Reference		
High (poorly differentiated or undifferentiated)	2.35	0.84–6.60	0.104
Lymph node involvement			
No	Reference		
Yes	2.63	0.61–11.28	0.19

## Discussion

SC is a kind of cutaneous adnexal carcinoma with low incidence, accounting for less than 1% of all malignant cutaneous neoplasms (4). Although SC most commonly involves the head and neck, especially the periocular area, it can occur anywhere sebaceous glands are located.

The precise etiology of SC is not well elucidated, but several factors have been implicated with an increased risk of development. Some cases are associated with inherited mutations in mismatch repair genes, such as Muir–Torre Syndrome (MTS), a form of Lynch syndrome, with microsatellite instability and cancer tendency (5, 6). In MTS, SC can occur concurrently with other cutaneous malignancies (keratoacanthomas) and internal malignancies (colorectal and genitourinary cancers), and, thus, additional screening workup is indicated (7). SC tends to affect older individuals, and the mean age of non-head and neck SC in this analysis was 67.7 years (8). However, SC may also present in younger patients, and the minimum age was 30 years in this cohort. For these patients, early screening for genetic mutations is important. Additionally, UV-light exposure is considered another risk factor in the development of SC (9). In this study, 96.4% of tumors involved sun-exposed skin of the trunk and extremities, and 91.2% of cases were diagnosed in whites, who were more susceptible to UV-induced damage. Sebaceous carcinoma exhibits a slight male preponderance, which was further confirmed in this analysis, with a male-to-female ratio of 1.98 (5). Moreover, prior radiation therapy and immunosuppression contribute to the development of SC (10–12).

Extraocular SC usually presents as an ulcerated yellowish nodule, resembling nonmelanoma skin cancers such as squamous cell carcinoma and basal cell carcinoma (13). Moreover, extraocular SC can also imitate benign conditions, including molluscum contagiosum, sebaceous cyst, pyogenic granuloma, or keratoacanthoma (14–16). Due to delayed visit and diagnosis and the large extension space for SC outside the head and neck, the tumor size tends to be large, with a mean of 2.2 cm in this analysis. Notably, histopathological examination, immunohistochemistry, and dermoscopic images may help detect SC and distinguish it from other easily confused diseases (17).

SC is usually indolent and with low-stage presentation. Nevertheless, there were cases of aggressive behavior and metastasis represented in the literature (18, 19). The metastasis rate was 1.6% in this cohort, and the 5-year OS was worse at 21.8%. Of 288 patients with a specific tumor grade, only eight were undifferentiated (2.8%), developing in the trunk (*n* = 5) and limbs (*n* = 2). Among undifferentiated carcinoma, three cases had distant disease, and all occurred in the trunk region. Therefore, there was a high incidence of distant metastasis in undifferentiated diseases (three of eight), especially those located in the trunk (three of five). In addition, patients with distant disease had a relatively large tumor size, a mean of 7 cm, ranging from 2 to 15.5 cm. It was concluded that although there were no distinct guidelines concerning systemic evaluation of SC, patients with undifferentiated disease, especially those in the trunk region, simultaneously with large tumors, may benefit from CT or MRI imaging to detect metastasis.

SC of the external genitalia is extremely rare, taking up 3.7% of the cohort. Multivariate analysis showed that SC on the external genitalia had a poorer outcome than those on the trunk, with a 3.3-fold increased risk of DSS, which indicated that the clinicopathologic behavior of SC on the external genitalia appeared different from other extraorbital sites. Notably, lymph node involvement and tumor grade have not been described as independent prognostic factors for SC outside the head and neck in this analysis.

Our study does have some shortcomings. Several variables, such as surgical modalities, histological types, metastatic sites, infiltration depth, genetic factors, tumor recurrence, and the time interval from onset of symptoms to diagnosis were not attained from the SEER database for further research. In addition, there was a large amount of missing data on the administration of radiation therapy and lymph node metastasis that may introduce a particular bias. Furthermore, the data included in this study were retrospective, and the conclusions drawn from this study still need to be verified by more high-quality clinical studies.

Despite the above limitations, this population-based study represents the first large-scale analysis of non-head and neck SC and provides significant insight into distant metastasis screening.

## Conclusion

This study conducted a comprehensive analysis of patients with non-head and neck SC from a well-defined population. Tumors located in these areas may grow to large sizes. In general, non-head and neck SC have a great prognosis, but survival becomes very poor once distant metastasis occurs. Patients with undifferentiated disease, especially those in the trunk region, are suggested to complete screening for distant metastasis.

## Data availability statement

The raw data supporting the conclusions of this article will be made available by the authors, without undue reservation.

## Ethics statement

Ethical approval was not required for the study involving humans in accordance with the local legislation and institutional requirements. Written informed consent to participate in this study was not required from the participants or the participants’ legal guardians/next of kin in accordance with the national legislation and the institutional requirements.

## Author contributions

XC: Writing – original draft, Software, Investigation, Conceptualization. YH: Writing – review & editing. MC: Writing – review & editing. JY: Writing – review & editing, Supervision.
